# Antibiotics and antibiotic-resistant bacteria in waters associated with a hospital in Ujjain, India

**DOI:** 10.1186/1471-2458-10-414

**Published:** 2010-07-13

**Authors:** Vishal Diwan, Ashok J Tamhankar, Rakesh K Khandal, Shanta Sen, Manjeet Aggarwal, Yogyata Marothi, Rama V Iyer, Karin Sundblad-Tonderski, Cecilia Stålsby- Lundborg

**Affiliations:** 1 Division of Global Health, (IHCAR), Department of Public Health Sciences, Karolinska Institutet, Nobels väg 9, SE 17177, Stockholm, Sweden; 2 Department of Public Health & Environment, RD Gardi Medical College, Agar Road, Ujjain, India; 3 Department of Environmental Medicine, RD Gardi Medical College, Agar Road, Ujjain, India; 4 Indian Initiative for Management of Antibiotic Resistance (IIMAR), N.G. Acharya and D.K. Marathe College, Chembur, Mumbai, India; 5 Shriram Institute for Industrial Research, 19, University Road, New Delhi, India; 6 Department of Microbiology, RD Gardi Medical College, Agar Road, Ujjain, India; 7 IFM Biology, section Ecology, Linköping University, Linköping, Sweden

## Abstract

**Background:**

Concerns have been raised about the public health implications of the presence of antibiotic residues in the aquatic environment and their effect on the development of bacterial resistance. While there is information on antibiotic residue levels in hospital effluent from some other countries, information on antibiotic residue levels in effluent from Indian hospitals is not available. Also, concurrent studies on antibiotic prescription quantity in a hospital and antibiotic residue levels and resistant bacteria in the effluent of the same hospital are few. Therefore, we quantified antibiotic residues in waters associated with a hospital in India and assessed their association, if any, with quantities of antibiotic prescribed in the hospital and the susceptibility of *Escherichia coli *found in the hospital effluent.

**Methods:**

This cross-sectional study was conducted in a teaching hospital outside the city of Ujjain in India. Seven antibiotics - amoxicillin, ceftriaxone, amikacin, ofloxacin, ciprofloxacin, norfloxacin and levofloxacin - were selected. Prescribed quantities were obtained from hospital records. The samples of the hospital associated water were analysed for the above mentioned antibiotics using well developed and validated liquid chromatography/tandem mass spectrometry technique after selectively isolating the analytes from the matrix using solid phase extraction. *Escherichia coli *isolates from these waters were tested for antibiotic susceptibility, by standard Kirby Bauer disc diffusion method using Clinical and Laboratory Standard Institute breakpoints.

**Results:**

Ciprofloxacin was the highest prescribed antibiotic in the hospital and its residue levels in the hospital wastewater were also the highest. In samples of the municipal water supply and the groundwater, no antibiotics were detected. There was a positive correlation between the quantity of antibiotics prescribed in the hospital and antibiotic residue levels in the hospital wastewater. Wastewater samples collected in the afternoon contained both a higher number and higher levels of antibiotics compared to samples collected in the morning hours. No amikacin was found in the wastewater, but *E.coli *isolates from all wastewater samples were resistant to amikacin. Although ciprofloxacin was the most prevalent antibiotic detected in the wastewater, *E.coli *was not resistant to it.

**Conclusions:**

Antibiotics are entering the aquatic environment of countries like India through hospital effluent. In-depth studies are needed to establish the correlation, if any, between the quantities of antibiotics prescribed in hospitals and the levels of antibiotic residues found in hospital effluent. Further, the effect of this on the development of bacterial resistance in the environment and its subsequent public health impact need thorough assessment.

## Background

Antibiotics are an important group of pharmaceuticals used in health care for the treatment and prevention of bacterial infections. Much of the antibiotic used in humans and animals remains unmetabolized and thus a significant amount is added to the environment via excretion. This ultimately contributes to the residues of antibiotics in recipient waters. Antibiotics might also be added to the environment from pharmaceutical plants and as a result of the dumping of unused antibiotics [1,2].

Besides antibiotics, resistant bacteria also enter into the aquatic environment from various sources [3]. It is not yet very clear whether the presence of antibiotics in the aquatic environment results in developing resistance in the bacteria found there. However, there is growing concern about the presence of antibiotic residues in the aquatic environment and their impact on resistance development, toxicity to aquatic communities and ultimately to public health [3-5].

Studies on antibiotic residues in hospital effluent and in other environmental niches have been conducted mostly in high-income countries, while studies in low- and middle-income settings are few and sparsely distributed [1,6-9] and only one study has estimated antibiotic residues in Indian hospital effluents [10]. Furthermore, concurrent studies on antibiotic prescription quantity in a hospital, antibiotic residue levels in its wastewater and resistant bacteria in the effluent of the same hospital are few [9,11,12].

The present study has quantified antibiotic residue levels in the incoming water and waste water (referred to as "hospital associated waters" or "water associated with the hospital" in the text) of a hospital in India and their association, if any, with quantities of antibiotics prescribed in the hospital and the antibiotic susceptibility of *Escherichia coli *(*E.coli*) found in the hospital effluent.

## Methods

### Settings and sub-studies

This cross-sectional study was conducted in the state of Madhya Pradesh in India. Specifically, the setting was the 500-bed teaching hospital attached to the R.D. Gardi Medical College, situated in a rural area near Ujjain city. Three sub-studies were conducted (i) quantification of antibiotics prescribed in the inpatient wards of the hospital (ii) estimation of antibiotic residues in hospital-associated waters and (iii) determination of susceptibility patterns in *E.coli *isolates obtained from the same waters. Further, the inter-relationship, if any, between the results obtained from these sub-studies was assessed.

### Selection of antibiotics

Seven antibiotics from four major antibiotic groups - amoxicillin (penicillins), ceftriaxone (cephalosporins), amikacin (aminoglycosides) and ofloxacin, ciprofloxacin, norfloxacin and levofloxacin (fluoroquinolones) - were selected for the study. The selection was based on the antibiotic prescription pattern in the inpatient wards of the hospital, the degree of metabolism of antibiotics in the human body, environmental stability and the known and suspected environmental impact of an antibiotic [1,13].

### Antibiotic prescription study

To estimate antibiotic consumption, information regarding antibiotics prescribed to patients around the same time as water sampling (6 days), was obtained from the hospital records. All the patients who were admitted during this period were included. The World Health Organization's (WHOs) Anatomical Therapeutic Chemical (ATC) classification index with defined daily doses (DDDs) was used to classify the antibiotics prescribed [14].

### Selection of sampling sites

Three sampling sites were selected, taking into account entry of safe water into the hospital system, its likelihood of becoming contaminated by antibiotics and exit of antibiotic-contaminated water into the environment. Samples were collected from (i) incoming safe water sources (Site 1) (ii) the point of exit of wastewater from inpatient wards of the hospital (Site 2) and (iii) 100 metres downstream from the hospital in subsequent drains (Site 3).

### Sampling protocol

Samples were collected twice- at 10:00 hrs and at 16:00 hrs. This was done as a safety measure as the dynamics of movement of antibiotics in the drains were unknown.

Grab samples (2250 ml) from each site were collected -two litres for analysis of antibiotic residues and 250 ml for analysis of antibacterial susceptibility - and were stored in an icebox at 4°C until they reached the analytical laboratories. Samples for antibiotic residue analysis were collected in screw-capped amber bottles wrapped in silver foil to prevent photo-degradation and were transported to the Shriram Institute for Industrial Research (SIIR) in New Delhi within 12 hrs. Samples for studying antibiotic susceptibility were collected in sterilized and paper-wrapped, 300-ml BOD bottles and were transferred immediately to the microbiology laboratory of the R.D. Gardi Medical College. In the chemical laboratory the samples were kept at - 20°C until analysis, while in the microbiology laboratory the analysis started immediately.

### Analysis of samples for the presence of antibiotic residues

The determination of the seven antibiotics in the hospital associated waters involved development and validation of a method [15], using liquid chromatography/tandem mass spectrometry (LC-MS/MS), (Waters 2695 series Alliance quaternary liquid chromatography system, Waters, USA, with a triple quadruple mass spectrometer, Quatro-micro API, Micromass, UK, equipped with electrospray interface and Masslynx 4.1 software (Micromass, UK) for data acquisition and processing). The instrument was operated in the multiple reaction monitoring positive ion mode. Nitrogen was used as a nebulization gas as well as dissolvation gas and was delivered at a flow rate of 50 l/hr and 750 l/hr respectively. Other typical MS settings are given in Table 1. Mass spectra were acquired over the scan range of m/z from 100 to 650 Dalton. For MS/MS mode, product-ion scan mass spectra of protonated molecules of the various antibiotics were acquired in the mass range of 50 to 600 Dalton. Two different characteristic fragments were monitored for each analyte to confirm the presence of the residues of various antibiotics. Table 2 shows the precursor ion and the daughter ions for the individual antibiotic. Quantification was done on the most intense ion transition.

**Table 1:** Mass spectrometric conditions used for determination of different antibiotics using LC-MS/MS

Antibiotic	Source temp (°C)	Dissolvation temp (°C)	Capillary voltage (Kv)	Cone voltage (V)	Collision energy	Dwell time (milli sec)
Amoxicillin	120	450	3	7	20	20
Ceftriaxone	120	450	3	20	15	20
Amikacin	120	450	3	20	15	20
Ofloxacin	120	450	3	22	22	20
Ciprofloxacin	120	450	3	20	30	20
Norfloxacin	120	450	3	20	35	20
Levofloxacin	120	450	3	20	25	20

**Table 2:** Data for retention time for different antibiotics, mass of the precursor ion monitored and mass of the daughter ions monitored

Antibiotic	Retention time (minutes)	Precursor ion [M + H] m/z	Daughter ion I m/z	Daughter ion II m/z
Amoxicillin	1.60	366.24	114	208
Ceftriaxone	1.78	555.25	324.14	396.2
Amikacin	1.16	586.48	425.43	264.19
Ofloxacin	1.05	362.29	318.22	261.13
Ciprofloxacin	1.08	333.2	231.05	245.03
Norfloxacin	1.02	320.2	231.02	204.6
Levofloxacin	1.1	362.22	261.13	318.24

The analysis was carried out on Waters X Terra MS C-18 column (2.1 mm × 100 mm, 5 μ particle size) using three different injections of each sample solution in three different solvent systems under isocratic conditions: (a) For norfloxacin, ofloxacin, ciprofloxacin and levofloxacin: A mixture (50:50 by volume) of solution of 0.1% formic acid in water and 0.1% formic acid in acetonitrile delivered at a flow rate of 0.2 ml/min; (b) For amikacin: A mixture (50:50 by volume) of solution of 0.1% formic acid in water and 0.1% formic acid in methanol delivered at a flow rate 0.2 ml/min and (c) For amoxicillin and ceftriaxone : A mixture (50:50 by volume) of solution of 0.1% formic acid in water and 0.1% formic acid in acetonitrile delivered at a flow rate 0.2 ml/min. The chromatographic run for each of the injections for all the antibiotics was completed within five minutes and the retention time for each analyte is mentioned in Table 2.

All solvents/chemicals used for the purpose were HPLC grade/analytical reagent grade. HPLC grade water, methanol and acetonitrile were purchased from Merck Specialities Private Limited, Mumbai, India, while acetic acid, formic acid, sulphuric acid, and triethylamine were purchased from S.D. Fine-Chem Limited, Mumbai, India.

### Preparation of sample solution for hospital associated water

Approximately 100 ml of accurately measured sample of the hospital associated water, filtered through 0.45-μ-filter membrane and acidified with sulphuric acid (pH 3.0) was loaded on an activated Waters Sep-Pak C18 cartridge (activated with 5 ml methanol, 5 ml methanol/water (50:50) followed by 5 ml acidified water at pH 3).

The cartridge was then washed with 5 ml acidified water and the adsorbed compounds were eluted with 5 ml eluant (5% solution of triethylamine in methanol). The eluant was evaporated off to almost dryness at 50°C using Nitrogen evaporator (Labconco Corporation, Kansas City, Missouri, USA). The dried extract was then dissolved in acetonitrile and the final volume made to 1 ml. This sample solution was injected thrice under three different mobile phase conditions (as given earlier) above for the different antibiotics.

### Preparation of standard solutions for calibration

Standard solutions for calibration were prepared with the following concentrations:

1.Combined calibration standard solutions for ofloxacin, ciprofloxacin, norfloxacin and levofloxacin at concentration levels of 2.5, 5.0, 10.0, 25.0, 50.0, 100.0, 250.0, 500.0 ng/ml respectively in the range 2.5 to 500 ng/ml.

2.Calibration standard solutions for amikacin at concentration levels of 50.0, 100.0, 250.0, 500.0 ng/ml respectively in the range 50 to 500 ng/ml.

3.Calibration standard solutions for ceftriaxone at concentration levels of 500.0, 1000.0, 1250.0, 2500.0 and 5000 ng/ml respectively in the range 500 to 5000 ng/ml.

4.Calibration standard solutions for amoxicillin at concentration levels of 100.0, 200.0, 500.0, 1000.0 and 1500.0 ng/ml respectively in the range 100 to 1500 ng/ml.

### Method Performance Characteristics

The method was validated as per the International Conference on Harmonisation (ICH) and the Eurachem guidelines [16,17]. Validation studies were performed for the various parameters i.e. linearity, precision (both interday and intraday), accuracy (percent recoveries), limit of detection, limit of quantification, specificity and selectivity. The results for the various validation parameters are tabulated in Table 3. All validation parameters were found well within the acceptable criteria of the guidelines.

**Table 3:** Summary of the validation parameters for the LC-MS/MS methods for the determination of residual antibiotics in water samples from a hospital in Ujjain, India

Antibiotics	**Correlation coefficient r**^**2**^	Coefficient of Variation (%RSD)		LOD μg/l	LOQ μg/l	% Recovery n = 5
		**Interday n = 6**	**Intraday n = 6**			

Amoxicillin	0.994	5.2	5.6	0.5	1.0	85 (%RSD 5.6)
Ceftrixone	0.997	2.1	1.8	2.5	5.0	84 (%RSD 4.1)
Amikacin	0.990	4.8	4.3	0.25	0.5	92 (%RSD 3.8)
Ofloxacin	0.999	3.1	2.57	0.01	0.025	95 (%RSD 3.7)
Ciprofloxacin	0.999	3.2	3.03	0.01	0.025	98 (%RSD 4.5)
Norfloxacin	0.999	3.4	3.02	0.01	0.025	90 (%RSD 3.2)
Levofloxacin	0.999	3.12	2.96	0.01	0.025	93 (%RSD 2.8)

LOD: Limit of detection; LOQ: Limit of quantification

### *E.coli *antibiotic susceptibility study

The estimation of total coliform group and fecal coliform group was done by determining the Most Probable Number (MPN) of bacteria in test samples, using standard multiple tube technique [18]. The test procedure included three phases, namely presumptive, confirmative & completed phase. For drinking water samples the presumptive phase consisted of, 5 tubes of 20 ml and 10 tubes containing 10 ml of lauryl tryptose broth medium. No dilutions were made for these samples. For wastewater samples, dilution were made with lauryl tryptose broth medium and 5 tubes for each dilution of 10 ml, 1 ml and 0.1 ml were used. All inoculated tubes were incubated at 35°C ± 0.5°C for 24-48 hrs. Tubes showing presence of growth (turbidity) with or without gas were submitted to confirmatory phase. All presumptive tubes showing positive results were gently shaken. Using sterile loop of 3 mm diameter, two loopful of culture was added to brilliant green lactose bile broth tubes and incubated at 35°C ± 0.5°C. Formation of gas within 48 hrs was taken as positive confirmation. Simultaneously EC broth tubes were also inoculated for completed phase and incubated at 44.5°C in water bath. Tubes showing acid and gas production within 24 hrs was taken as positive result indicating presence of fecal coliforms in the samples. Further, *E.coli *were confirmed by sub culturing on MacConkey agar to obtain pure culture. Following biochemical confirmation by standard biochemical tests, confirmed *E. coli *isolates were tested for susceptibilities using the standard Kirby Bauer disc diffusion method for seven antibiotics according to the Clinical and Laboratory Standard Institute (CLSI) breakpoints [19]. After matching turbidity of the inoculum with McFarland 0.5 standard, the cotton swab dipped into inoculum was inoculated on the Muller-Hinton agar. The antibiotic discs were placed on the inoculated plates and then incubated at 35°C ± 0.5°C for about 18-20 hours. The diameter (in millimeters) of the zone of inhibition was measured and interpreted as resistant, intermediate or sensitive using CLSI guidelines for the respective antibiotic [19]. Reference strain ATCC 25922 of *E. coli *was used for quality control.

All the chemicals and media used were of standard grade and procured from HiMedia Laboratories Private Limited, Mumbai, India.

### Data management and statistical analysis

Data on antibiotic prescription, antibiotic residue levels and antibiotic susceptibility analysis was entered and analysed in SPSS 17 [20]. The results of prescription data are presented as the total amount of antibiotics prescribed in milligrams (mg) during the six days and number of defined daily doses (DDDs) [14]. The amount of antibiotic residue levels is presented in μg/l. To determine the relationship between quantities of antibiotic prescribed and antibiotic residue levels obtained in hospital wastewater, Spearman rank correlation was performed.

## Ethical approval

Approval for the study was obtained from the ethical committee of the R.D. Gardi Medical College, Ujjain.

## Results

Prescription studies showed that ciprofloxacin was the most commonly prescribed antibiotic (Table 4).

**Table 4:** Prescribed quantities of targeted antibiotics, and their respective DDDs in inpatient wards of a hospital in Ujjain, India

Antibiotics	ATC code	Quantity of prescribed antibiotics/6 days (in mg)	No. of patients to whom antibiotic was prescribed	DDD (mg)	No of DDDs
**Penicillins**					
Amoxicillin	J01CA04	19150	4	1000	19.15

**Cephalosporins**					
Ceftriaxone	J01DD04	126700	24	2000	63.4

**Amino glycosides**					
Amikacin	J01GB06	63825	26	1000	63.8

**Fluoroquinolones**					
Ofloxacin	J01MA01	2700 (O)	4	400	6.8
		4200 (P)		400	10.5
Ciprofloxacin	J01MA02	134700 (O)	66	1000	134.7
		29000 (P)		500	58.0
Norfloxacin	J01MA06	47600 (O)	23	800	59.5
Levofloxacin	J01MA12	3000 (O)	2	500	6

O = orally administered, P = parenterally administered

The results of the antibiotic residue studies are summarized in Table 5. These studies showed that in samples from the municipal water supply and the groundwater, no antibiotics were present. Four of the 7 antibiotics studied were detected in the analysed wastewater samples. All the detected antibiotics were from the fluoroquinolone group, the highest concentration being that of ciprofloxacin (236.6 μg/l). Samples collected from site 3 located 100 metres from the hospital showed a higher number of antibiotics and also higher residue levels than the site closer to the hospital (Site 2). Samples collected in the afternoon (16:00) showed a relatively higher number of antibiotics and also higher residue levels of antibiotics compared to samples collected in the morning hours (10:00). Spearman's rank correlation was positive (r = 0.29; p = 0.51) between antibiotic prescription data and antibiotic residue levels in hospital wastewater (all the antibiotics). When only antibiotics from the fluoroquinolone group were examined, correlation was positive and higher (r = 0.80; p = 0.2).

**Table 5:** Levels of monitored antibiotics (μg/l) in waters associated with a hospital in Ujjain, India using LC-MS/MS

Antibiotics	Site 1		Site 2		Site 3	
	
	Ground Water	Municipal Water	At 10:00	At 16:00	At 10:00	At 16:00
Amoxicillin	--	--	--	--	--	--
Ceftriaxone	--	--	--	--	--	--
Amikacin	--	--	--	--	--	--
Ofloxacin	--	--	--	4.5	5.6	7.5
Ciprofloxacin	--	--	2.2	218.3	67.3	236.6
Norfloxacin	--	--	--	6.4	29.6	22.8
Levofloxacin	--	--	--	5.0	6.8	8.8

-- = Below Detection Limit

Site 1 = Incoming safe water (received in hospital only once a day)

Site 2 = At the point of exit of inpatient wards of the hospital

Site 3 = 100 metres from the hospital in subsequent drains

No *E.coli *were detected in the municipal water supply, however, *E.coli *were found in the groundwater and showed resistance to ciprofloxacin, intermediate resistance to amikacin and sensitivity to the other antibiotics tested (Table 6). *E.coli *from all wastewater samples were resistant to amikacin and sensitive to amoxicillin, with only *E.coli *from site 2 showing intermediate resistance to ciprofloxacin. Thus, no specific relationship between *E.coli *susceptibility/resistance and antibiotic prescription and/or residue level in wastewater was discernible.

**Table 6:** Antibiotic susceptibility of *E.coli *found in samples taken from waters associated with a hospital in Ujjain, India

	Disc Concentration (μg)	Site 1*	Site 2		Site 3	
		
		Ground Water	At 10:00	At 16:00	At 10:00	At 16:00
Amoxicillin	30	S	S	S	S	S
Ceftriaxone	30	S	S	S	I	I
Amikacin	30	I	R	R	R	R
Ofloxacin	5	S	I	S	S	S
Ciprofloxacin	5	R	I	I	S	S
Norfloxacin	10	S	S	S	S	I
Levofloxacin	5	S	S	S	I	I

R= Resistant; I= Intermediate; S= Sensitive

Site 1 = Incoming safe water (received in hospital only once a day) *No *E. coli *was detected in the municipal water supply.

Site 2 = At the point of exit of inpatient wards of the hospital.

Site 3 = 100 metres from the hospital in subsequent drains.

## Discussion

To our knowledge, this is the first study in India, which has attempted to quantify concurrently, in the same hospital, consumption of antibiotics, estimation of antibiotic residues in hospital-associated waters and antibiotic sensitivity amongst *E.coli *found in these waters.

From this study, it appeared that some of the antibiotics prescribed to the patients in hospitals are reaching the nearby aquatic environment. There was a positive, (though not statistically significant) correlation between antibiotic prescription quantity and residue levels in hospital effluent. Specifically for fluoroquinolones, the correlation was high. The reason for it not being statistically significant could be the small sample size in this study. Watkinson et al [21] also showed that the presence of antibiotics in hospital effluent depended on the volume of antibiotic prescription. They also found a positive correlation, which was not statistically significant. There are factors other than prescription volume that can play an important role in the quantities of antibiotics detected in the hospital effluent like the metabolism of antibiotics, their stability in the environment, temperature, water flow rate, time of sample collection, matrix effect, etc [1,5].

Antibiotics from the fluoroquinolone group have also been found in hospital wastewater in other parts of the world. Amongst fluoroquinolones, ciprofloxacin was the most commonly found antibiotic in hospital effluent [1,8-11,22-24]. High residue levels of fluoroquinolones in the aquatic environment can cause genotoxic effects and can modify bacterial strains like *Salmonella typhimurium *at a residue level as low as 5 μg/l for norfloxacin and 25 μg/l for ciprofloxacin [25]. In our study, the concentration of norfloxacin (6.4-29.6 μg/l) and ciprofloxacin (2.20-236.6 μg/l) in the drains was high, and this is a matter of concern in terms of its wider public health impact.

Ceftriaxone residue was not detected in any of the samples analyzed, despite the fact that it was one of the major antibiotics prescribed in the hospital. Neither have ceftriaxone and other antibiotics with the β -lactam ring generally been detected in hospital effluent elsewhere. Reasons for their non-detection have been cited as the easy degradation of the β -lactam ring, its high metabolic rate and the process of decarboxylation [1].

Results from the sampling repeated at the same point indicated that there were considerable variations in antibiotic concentrations detected at the same place over time. The residue levels of all the antibiotics from the fluoroquinolone group were higher at 16:00 hrs than at 10:00 hrs. Lindberg et al. [22] also reported considerable variation in residue levels when sampling was done at various times of the day at the same place. Such temporal variations might arise due to variations in the administration of antibiotics during the course of the day, water usage (and hence wastewater flow), as well as various pharmacokinetic factors such as metabolism, half-life and excretion rate [1,5]. In addition, other processes such as sorption, hydrolysis and photolysis may influence the concentrations detected in wastewater at any particular time. In general, however, very little information is available on the temporal, spatial and diurnal behaviour of antibiotics in the aquatic environment. Longitudinal sampling strategies like monitoring of hospital effluents for antibiotic residues over several days with recording also of flow characteristics is needed to assess the potential risks associated with the release of these residues on environment in general and resistance in particular.

Several studies have documented the presence of antibiotic-resistant bacteria in the aquatic environment in general and hospital effluent in particular [3]. It is not yet clearly established that the mere presence of antibiotic residues in the aquatic environment by itself influences the development of resistance in natural bacterial communities. It has been suggested that other factors like the density of resistant bacteria, duration of exposure to the antibiotic and presence of a favourable environment need to be considered as well [3]. In our study, amikacin residue was not detected in any of the analyzed samples but nevertheless, *E. coli *from all the wastewater samples showed resistance to this antibiotic and even *E.coli *isolated from ground water displayed an intermediate behaviour. In contrast, none of the *E.coli *isolated from the wastewater samples was resistant to ciprofloxacin but showed intermediate behaviour in three samples, though high levels of ciprofloxacin residue were detected in the wastewater.

Antibiotics have been detected earlier in hospital effluent in high-income countries [3]. The present study has highlighted the fact that antibiotics also enter the aquatic environment through hospital effluent in low- and middle-income countries. Unlike high-income countries, the situation can become more problematic in low- and middle-income countries, where resource constraints might result in untreated wastewater. In India, for example, not much wastewater undergoes any type of treatment and along with other pollutants; antibiotics must also be finding their way into recipient waters like rivers, lakes, reservoirs, etc. In situations where the wastewater is not subjected to any treatment, it is sometimes argued that antibiotics are diluted in the recipient waters in comparison with therapeutic concentrations and may not cause any harm. However, it is widely recognized that exposure to sub-therapeutic concentrations over long periods of time provides ideal conditions for the transfer of resistance genes. Use of non-culture techniques is important to understand the dynamics of transfer of antibiotic resistance genes. This was not possible in the present study, due to limited resources. However, we plan to use these techniques in future studies.

As regards the presence of resistant bacteria in the wastewater, treatment systems can be a way of combating this problem, as it has been shown that resistant bacteria are eliminated relatively efficiently in wastewater treatment plants [3]. Further, from the point of view of low- and middle-income countries, low-cost, environment-friendly techniques could be designed and developed for the removal of antibiotics from wastewater using methods developed to simultaneously remove resistant bacteria. This may offer a comprehensive solution to the problem of antibiotic resistance development in the aquatic environment. We expect that the results of this study will be helpful in creating a basis for further scientific enquiry in India and other countries regarding the presence of emerging contaminants like antibiotics in environment and its impact on public health.

## Conclusions

Ciprofloxacin was the most common antibiotic prescribed in the studied hospital, and the concentration of this compound was highest both in the hospital wastewater and in the downstream drain system. The antibiotic residue levels were above the levels that can cause gentoxic effects and modify bacterial strains. Samples collected in the afternoon contained both a higher number and higher levels of the antibiotics compared to samples collected in the morning hours. There was a positive correlation between quantities of antibiotics prescribed in the hospital and their residue levels in the hospital wastewater. No antibiotics were detected in the municipal water supply or in samples from the ground water supply, which was a redeeming feature of the study.

Although, ciprofloxacin was the most prevalent antibiotic found in wastewater, *E.coli *from wastewater was not resistant to it but showed intermediate behaviour in three samples. In contrast, no amikacin was found in the wastewater, but *E.coli *isolated from all wastewater samples showed resistance to amikacin. More in-depth studies are needed to assess the correlation between the quantity of antibiotics prescribed and both the levels of antibiotic residues in hospital effluent and the effects on the development of bacterial resistance in the environment.

## Competing interests

The authors declare that they have no competing interests.

## Authors' contributions

VD initiated the concept, coordinated the study, carried out the fieldwork, analysed the results and drafted the manuscript. AJT participated in developing the concepts, participated in the design and coordination of the study and in the analysis and interpretation of results and participated in drafting and finalising the manuscript. RKK, SS and MA were responsible for developing methods as well as analyzing antibiotics. YM and RVI planned and carried out the bacterial susceptibility study. KST participated in the development of the sampling plan in the initial stages of the study and gave input from time to time. CSL participated in developing the concept, participated in the design and coordination of the study and participated in the analysis and interpretation of results and in drafting and finalising the manuscript. All authors have read and approved the final manuscript.

## Pre-publication history

The pre-publication history for this paper can be accessed here:

http://www.biomedcentral.com/1471-2458/10/414/prepub
